# A N-terminal truncated intracellular isoform of matrix metalloproteinase-2 impairs contractility of mouse myocardium

**DOI:** 10.3389/fphys.2014.00363

**Published:** 2014-09-25

**Authors:** David H. Lovett, Charles Chu, Guanying Wang, Mark B. Ratcliffe, Anthony J. Baker

**Affiliations:** ^1^Cardiology Division, San Francisco Veteran Affairs Medical CenterSan Francisco, CA, USA; ^2^Department of Medicine, University of California, San FranciscoSan Francisco, CA, USA; ^3^Department of Surgery, University of California, San FranciscoSan Francisco, CA, USA; ^4^Joint UC Berkeley/UCSF Bioengineering GroupSan Francisco, CA, USA

**Keywords:** contraction, heart failure, myofilament, Ca^2+^ transient, remodeling, MMP-2

## Abstract

The full-length isoform of matrixmetalloproteinase-2 (FL-MMP-2) plays a role in turnover of the cardiac extracellular matrix. FL-MMP-2 is also present intracellularly in association with sarcomeres and, in the setting of oxidative stress, cleaves myofilament proteins with resultant impaired contractility. Recently, a novel N-terminal truncated MMP-2 isoform (NTT-MMP-2) generated during oxidative stress was identified and shown to induce severe systolic failure; however, the injury mechanisms remained unclear. In this study, cardiac-specific NTT-MMP-2 transgenic mice were used to determine the physiological effects of NTT-MMP-2 on: force development of intact myocardium; the function of cardiac myofilaments in demembranated myocardium; and on intracellular Ca^2+^ transients in isolated myocytes. We related the contractile defects arising from NTT-MMP-2 expression to the known intracellular locations of NTT-MMP-2 determined using immunohistochemistry. Comparison was made with the pathophysiology arising from cardiac-specific FL-MMP-2 transgenic mice. Consistent with previous studies, FL-MMP-2 was localized to myofilaments, while NTT-MMP-2 was concentrated within subsarcolemmal mitochondria and to sites in register with the Z-line. NTT-MMP-2 expression caused a 50% reduction of force development by intact myocardium. However, NTT-MMP-2 expression did not reduce myofilament force development, consistent with the lack of NTT-MMP-2 localization to myofilaments. NTT-MMP-2 expression caused a 50% reduction in the amplitude of Ca^2+^ transients, indicating impaired activation.

**Conclusions:** Unlike FL-MMP-2, NTT-MMP-2 does not mediate myofilament damage. Instead, NTT-MMP-2 causes impaired myocyte activation, which may involve effects due to localization in mitochondria and/or to transverse tubules affecting Ca^2+^ transients. Thus, FL-MMP-2 and NTT-MMP-2 have discrete intracellular locations and mediate different intracellular damage to cardiac myocytes.

## Introduction

While the overall mortality rates for acute myocardial infarction have decreased in recent years due to the advent of more effective acute interventions, the incidence of dysfunctional post-myocardial ventricular remodeling continues to increase. The most serious complication of post-infarction remodeling is congestive heart failure, which has become one of the leading causes of death in ischemic cardiac disease. Recent experimental and clinical evidence has underscored a critical role for matrix metalloproteinase-2 (MMP-2) in ischemic cardiac disease, including post-infarction ventricular remodeling (Alfonso-Jaume et al., 2006; Mukherjee et al., 2006; Bergman et al., 2007; Zhou et al., 2007; Nilsson et al., 2011; Lovett et al., 2012, 2013; Cogni et al., 2013). Our current gaps in knowledge are substantial and relate to an incomplete understanding of the multiple pathophysiologic roles of MMP-2 in post-infarction remodeling.

MMP-2 affects multiple factors involved in ischemic ventricular remodeling, inflammation and cardiomyocyte apoptosis. MMP-2 was originally investigated as the full-length secreted enzyme involved in the turnover of the extracellular matrix. However, recent studies have defined a pool of intracellular MMP-2 arising from a fraction of full-length MMP-2 (FL-MMP-2) that escapes from the secretory pathway and, in the setting of redox stress, causes intracellular damage to the myofilaments in cardiomyocytes (Wang et al., 2002; Sawicki et al., 2005; Schulz, 2007; Lovett et al., 2012, 2013).

Under pathophysiological conditions, MMP-2 is known to be associated with cardiac dysfunction. To determine the role of MMP-2 in the absence of superimposed injury, we previously used cardiac-specific transgenic mice that expressed constitutively active FL- MMP-2 (Wang et al., 2006; Bergman et al., 2007). We reported that FL-MMP-2 expression resulted in a 50% reduction of contractile force of electrically stimulated cardiac trabeculae. Furthermore, impaired force development was associated with a 50% reduction of myofilament force development assessed using demembranated myocardium exposed to activating solutions (Wang et al., 2006). These studies suggest that, in the absence of superimposed injury, FL-MMP-2 impairs myocardial contraction by damaging the myofilaments. Consistent with this, FL-MMP-2 localizes to the myofilaments (Bergman et al., 2007) and recent studies demonstrate that intracellular FL-MMP-2 cleaves several myofilament-associated proteins, including troponin I, titin, α-actinin and myosin essential light chain (Ali et al., 2011).

We recently identified a second intracellular MMP-2 isoform, N-terminal truncated MMP-2 (NTT-MMP-2) that is generated by redox stress-mediated activation of an alternate promoter in the first intron of the MMP-2 gene (Lovett et al., 2012). Oxidative stress-mediated generation of NTT-MMP-2 may contribute to progressive cardiac dysfunction in the setting of ischemia (Lovett et al., 2012). Translation of the NTT-MMP-2 transcript is initiated at Methionine^77^, resulting in an N-terminal truncated MMP-2 protein lacking the secretory sequence and the inhibitory prodomain. Thus, the NTT-MMP-2 protein remains intracellular and is enzymatically active. *In-vitro* cell fractionation studies localized the NTT-MMP-2 isoform to cytosolic and mitochondrial fractions (Lovett et al., 2012). Within the context of the intact heart, the NTT-MMP-2 isoform was primarily localized to mitochondria and also localized to perpendicular arrays across the long axis of individual cardiomyocytes (Lovett et al., 2013). In addition to activation of NTT-MMP-2, ischemic cardiac injury involves activation of multiple other factors. Therefore, for the current study, to focus only on the physiological effects mediated by NTT-MMP-2, we used transgenic expression of NTT-MMP-2 in mouse hearts. Cardiac-specific transgenic expression of the NTT-MMP-2 isoform induced severe systolic failure; however, the injury mechanisms involved were unclear (Lovett et al., 2013). Therefore, the goal of this study was to investigate the mechanisms of NTT-MMP-2-mediated cardiac systolic failure. We report that the NTT-MMP-2 isoform directly impairs cardiomyocyte contractility by affecting calcium handling. Thus, the FL-MMP-2 and NTT-MMP-2 isoforms contribute differently to cardiac contractile dysfunction.

## Materials and methods

### Transgenic mice

This institution is accredited by the American Association for the Accreditation of Laboratory Animal Care (Institutional PHS Assurance Number is A3476-01). The investigation was approved by the Animal Care and Use Subcommittee (IACUC) of the San Francisco Veterans Affairs Medical Center (protocol 09-053-03) and conformed to the *Guide for the Care and Use of Laboratory Animals* published by the National Institutes of Health (Revised 2011).

Cardiac-specific transgenic mice expressing the NTT-MMP-2 isoform were generated in the CD-1 background as previously described (Lovett et al., 2013). Four to five month old transgenic and littermate controls were used. Cardiac-specific transgenic mice expressing the FL-MMP-2 isoform were generated and characterized as reported (Bergman et al., 2007). Four to five month old transgenic mice and littermate controls were used.

### Immunohistological detection of MMP-2 isoforms

For localization of the c-myc-tagged FL-MMP-2 transgene, paraformaldehyde-fixed ventricular sections were incubated with murine monoclonal anti-c-myc (9E11, Abcam, Cambridge, MA), followed by the M.O.M. kit (Vector, Burlingame, CA) and development with VIP purple substrate (Vector) as previously described (Bergman et al., 2007).

Intracellular localization of the NTT-MMP-2-EGFP transgene was performed on deparaffinized sections following citrate antigen retrieval (Vector) using a 1:1000 dilution of rabbit polyclonal anti-GFP (Abcam, Ab6556) for 4 h, followed by incubation with 1:2000 biotinylated goat-anti-rabbit IgG. Histochemical development with diaminobenzidine-Ni^+2^ (DAB- Ni^+2^, Vector) was restricted to 3 min to limit reaction product migration, followed by a hematoxylin counterstain. Images were digitally captured at high resolution (4000 dpi) using a Zeiss Nomarksy optics microscope. A dense focus of DAB- Ni^+2^ reaction product in the transgenic image was sampled in Adobe Photoshop CS4. Foci with similar pixel densities were converted to a fluorescent pseudocolor using the Image Adjust/Change Color tool to enhance contrast with background DAB- Ni^+2^ staining. The wild type control images were processed simultaneously in an identical fashion.

### Contraction of intact myocardium

Mice were deeply anesthetized with pentobarbital (100 mg/kg i.p.) mixed with heparin (100 U). Hearts were removed from anesthetized mice and flushed with modified Krebs-Henseleit, as previously described (Wang et al., 2006). A free-running trabecula was dissected from the right ventricle. For NTT-MMP-2 mice and wild type controls, the width, thickness and cross-sectional area of trabeculae were: 148 ± 62 μm, 79 ± 16 μm, 0.012 ± 0.007 mm^2^, *n* = 4; and 174 ± 11 μm, 123 ± 18 μm, 0.017 ± 0.003 mm^2^, *n* = 4; there was not a significant difference in these values between NTT-MMP-2 and wild type (*P* > 0.05, ANOVA). Trabeculae were placed in a muscle chamber and mounted to a force transducer, and sarcomere length set to 2.1 μm. Trabeculae were superfused with Krebs-Henseleit solution at 22°C and field stimulated (0.5 Hz pacing frequency and voltage 1.5 × threshold) (Wang et al., 2006).

### Assessment of myofilament contraction

After recording electrically stimulated contractions, these same trabeculae were demembranated using 1% Triton X-100, and *in-vitro* myofilament function was assessed using steady state contractions at various bath [Ca^2+^] as previously described (Wang et al., 2006). The relationship between steady state force (F) and [Ca^2+^] was fit to the Hill equation: F = F_max_ × [Ca^2+^]^*n*_H_^/([Ca^2+^]^*n*_H_^ + EC_50_^*n*_H_^), where F_max_ is the maximum Ca^2+^-activated force, EC_50_ is the [Ca^2+^] at which F is 50% of F_max_, and *n*_H_ is Hill coefficient reflecting the slope of the Ca^2+^-force relationship at EC_50_.

### Measurement of Ca^2+^ transients

Myocyte Ca^2+^ transients were recorded using from the fluorescence of Fura-2 as we recently described (Chu et al., 2013). Briefly, mice were deeply anesthetized with pentobarbital (100 mg/kg i.p.) mixed with heparin (100 U). Hearts were rapidly removed and immersed in ice-cold arrest solution (in mM: NaCl 120, KCl 30, CaCl_2_ 0.1). Myocytes were isolated by enzymatic digestion of the heart by retrograde perfusion of the aorta with collagenase solution (O'Connell et al., 2007). After collagenase treatment, the heart was placed in a “stop buffer” to halt enzymatic digestion (O'Connell et al., 2007). The heart was placed in 10 mL of stop buffer and gently teased with fine forceps followed by repeated pipetting to release the cells. The cell isolation buffers contained 10 mM BDM (2,3-Butanedione monoxime) to prevent cell contraction. Cells were used within 4 h of isolation. We studied 38 cells from 9 animals.

Myocytes were loaded with the Ca^2+^ indicator Fura-2 by exposure to 1 μM Fura-2-AM for 20 min. After washing, myocytes were equilibrated for 20 min to allow for de-esterification of the indicator and used within 1.5 h. after completion of the loading protocol.

Myocytes were superfused in a small glass-floored chamber with Krebs Henseleit solution containing (in mM): NaCl, 112; KC1, 5; MgCl_2_, 1.2; glucose, 10; NaHCO_3_, 24; Na_2_SO_4_, 1.2; NaH_2_PO_4_, 2.0; and CaCl_2_, 1. The perfusate was oxygenated with 95% O_2_/5% CO_2_ to give a pH of 7.4 at 22°C. The chamber was mounted on an inverted Nikon Diaphot microscope and cells visualized at x40 magnification. Cells were electrically stimulated with 4 ms square wave pulses, at a frequency of 0.5 Hz. Cell contraction and Fura-2 fluorescence were monitored using an IonOptix Hyperswitch system (Milton, MA). Cell contraction was computed by monitoring changes in muscle sarcomere length measured from the myocyte striation spacing. Fura-2 fluorescence at an emission wavelength of 510 nm was measured, with the excitation alternated between 340 and 380 nm at a frequency of 240 Hz. Background autofluorescence at each wavelength was subtracted before computing the Fura-2 fluorescence ratio.

### Statistical analysis

Data presented as mean ± SE. Statistical comparison used unpaired Student's *t*-test, and Two-Way ANOVA with repeated measures followed by the Bonferroni's *post-hoc* test to compare between groups. The significance level was set at *P* < 0.05.

## Results

### Distinct localization of NTT-MMP-2 vs. FL-MMP-2

**Figure 1:**
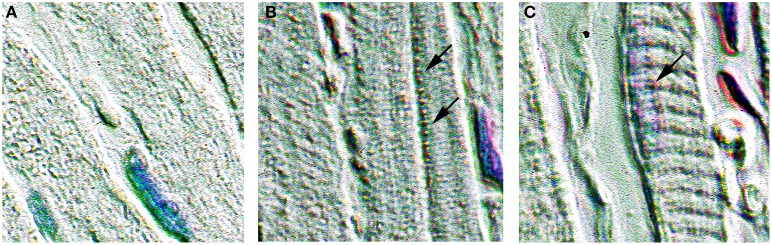
**Expression of NTT-MMP-2 in transgenic hearts**. Immunohistochemical staining for the eGFP epitope tag present on the NTT-MMP-2 transgene. Pseudo-color enhanced immunohistochemistry of wild type **(A)** and transgenic **(B,C)** ventricular sections (400× original magnification). NTT-MMP-2 is present within mitochondria, particularly within subsarcolemmal mitochondria (indicated by arrows in **B**) and localizes to clusters in register with the Z-line (arrow in **C**). No NTT-MMP-2 staining pattern was evident in wild type hearts. Intracellular NTT is much less abundant that the FL-MMP-2 isoform, therefore, pseudo-color enhancement was used to improve contrast and to visualize the subcellular localization.

Figure 1 shows immunohistochemical staining of ventricular myocardium from NTT-MMP-2 transgenic mice. Punctate staining for eGFP-tagged NTT-MMP-2 transgene is concentrated within subsarcolemmal mitochondria (Figure 1B) consistent with our previous report (Lovett et al., 2013). In addition, punctate staining for the NTT-MMP-2 transgene is localized to discrete clusters that are in register with the Z-line of the sarcomere (Figure 1C), which may reflect structures associated with transverse tubules. This is consistent with our previous finding of NTT-MMP-2 localized to perpendicular arrays across the long axis of individual cardiomyocytes (Lovett et al., 2013). Myocardium from wild type mice did not show specific localization of signal for NTT-MMP-2 (Figure 1A).

**Figure 2:**
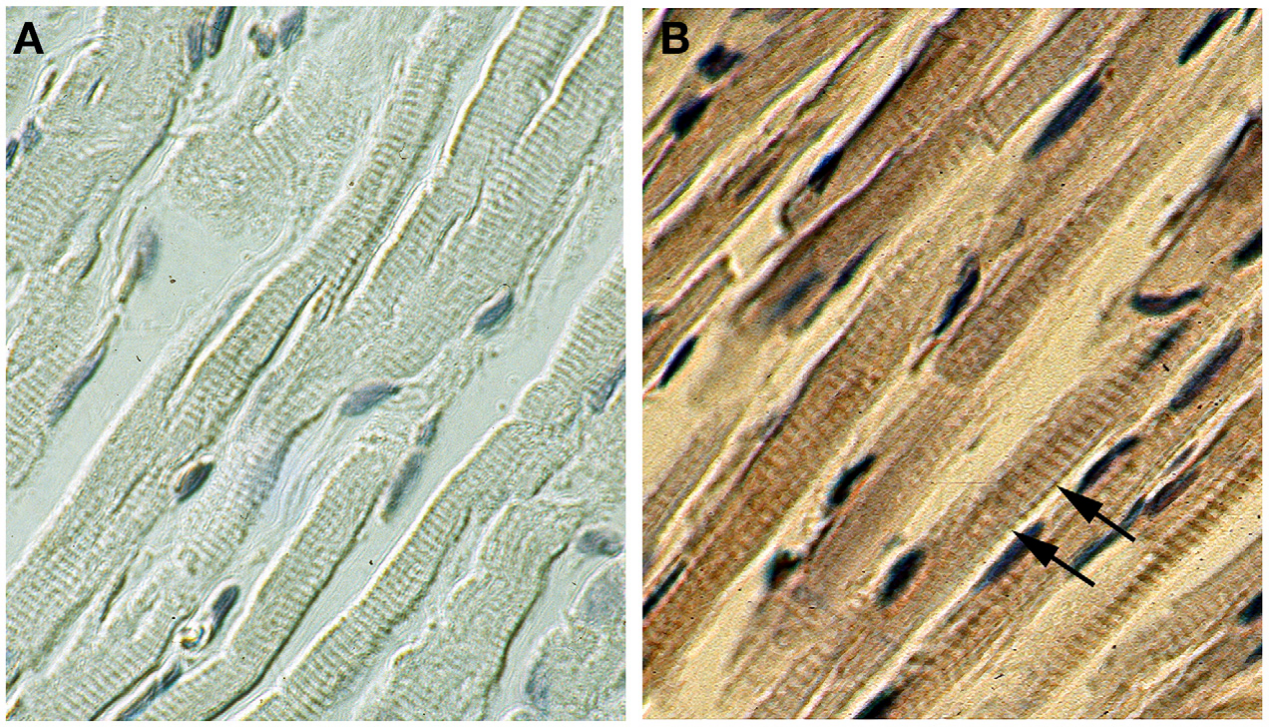
**Expression of FL-MMP-2 in transgenic hearts**. Immunohistochemical staining for the c-myc epitope tag of the FL-MMP-2 transgenic using peroxidase stain for wild type **(A)** and transgenic **(B)** ventricular sections (400× original magnification). The staining in a regular banding pattern (indicated by arrows in **B**) is consistent with a sarcomeric localization. No staining was evident in wild type.

Figure 2 shows immunohistochemical staining of ventricular myocardium from FL-MMP-2 transgenic mice. Consistent with our previous report (Bergman et al., 2007), a prominent striated pattern is evident in the stained section consistent with a sarcomeric localization of the c-myc tagged FL-MMP-2 transgene (Figure 2B). Ventricular myocardium from wild type mice did not show significant staining for the FL-MMP-2 transgene (Figure 2A).

These findings confirm that the NTT-MMP-2 and FL-MMP-2 isoforms were localized to distinct intracellular compartments.

### Transgenic NTT-MMP-2 expression reduced myocardial force development

**Figure 3:**
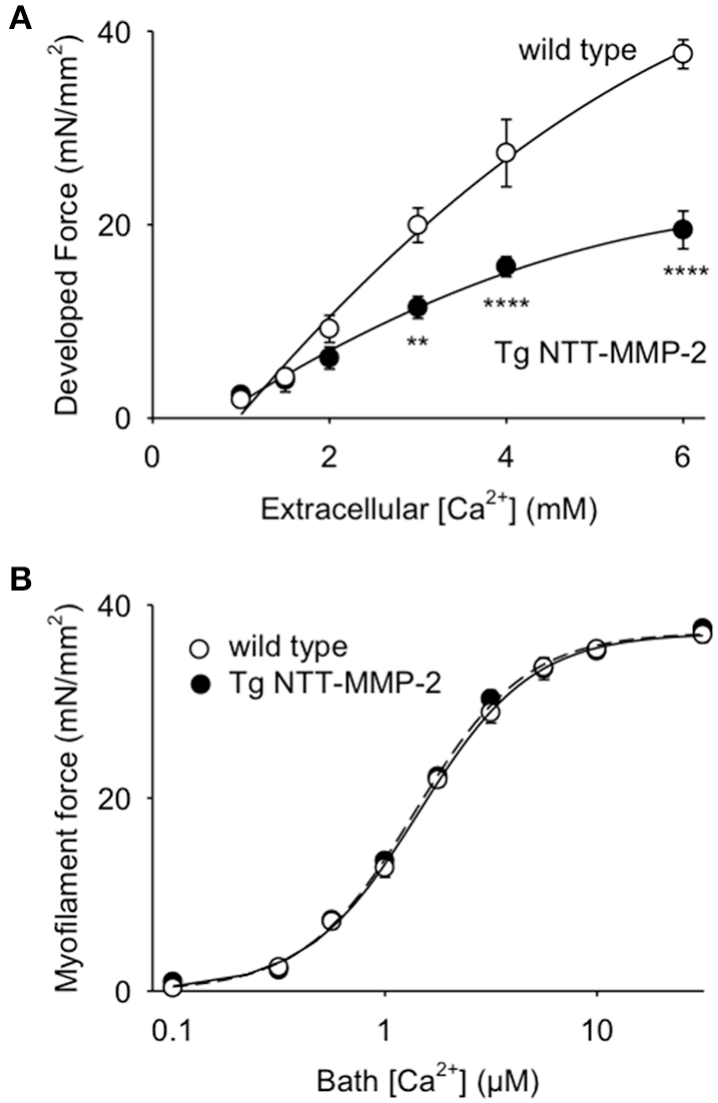
**(A)** Relationship between developed force vs. bath [Ca^2+^] for electrically stimulated intact trabeculae. Trabeculae from transgenic (Tg) NTT-MMP-2 mice developed less force with raised bath [Ca^2+^] compared to wild type controls (*P* < 0.001, ANOVA; mean ± SE, *n* = 4/group, ^**^P < 0.01, ^****^P < 0.0001). **(B)** Relationship between steady-state force development and [Ca^2+^] for skinned trabeculae (error bars were less than the symbol size). There was no statistical difference in myofilament force development of trabeculae from wild type vs. Tg NTT-MMP-2 mice (Two-Way ANOVA), thus, expression of NTT-MMP-2 did not reduce myofilament force in skinned myocardium. Lines show fitting of the pooled data to the Hill equation.

Figure 3A shows electrically stimulated force development of myocardium from wild type and NTT-MMP-2 transgenic hearts. Extracellular bath [Ca^2+^] was varied to assess force development over the full range of cardiac muscle activation. Figure 3A shows considerably lower force development by intact myocardium from NTT-MMP-2 transgenic mice compared to wild type mice, at the higher levels of activation. Diastolic force levels were low, with no difference in diastolic force level between NTT-MMP-2 transgenic vs. wild type myocardium (0.79 ± 0.25 vs. 0.76 ± 0.22 mN/mm^2^, *P* > 0.05).

Thus, both transgenic NTT-MMP-2 expression, and transgenic FL-MMP-2 expression (Wang et al., 2006) cause similar reductions of force development by intact myocardium.

### Transgenic NTT-MMP-2 expression did not affect myofilament force development

**Figure 4:**
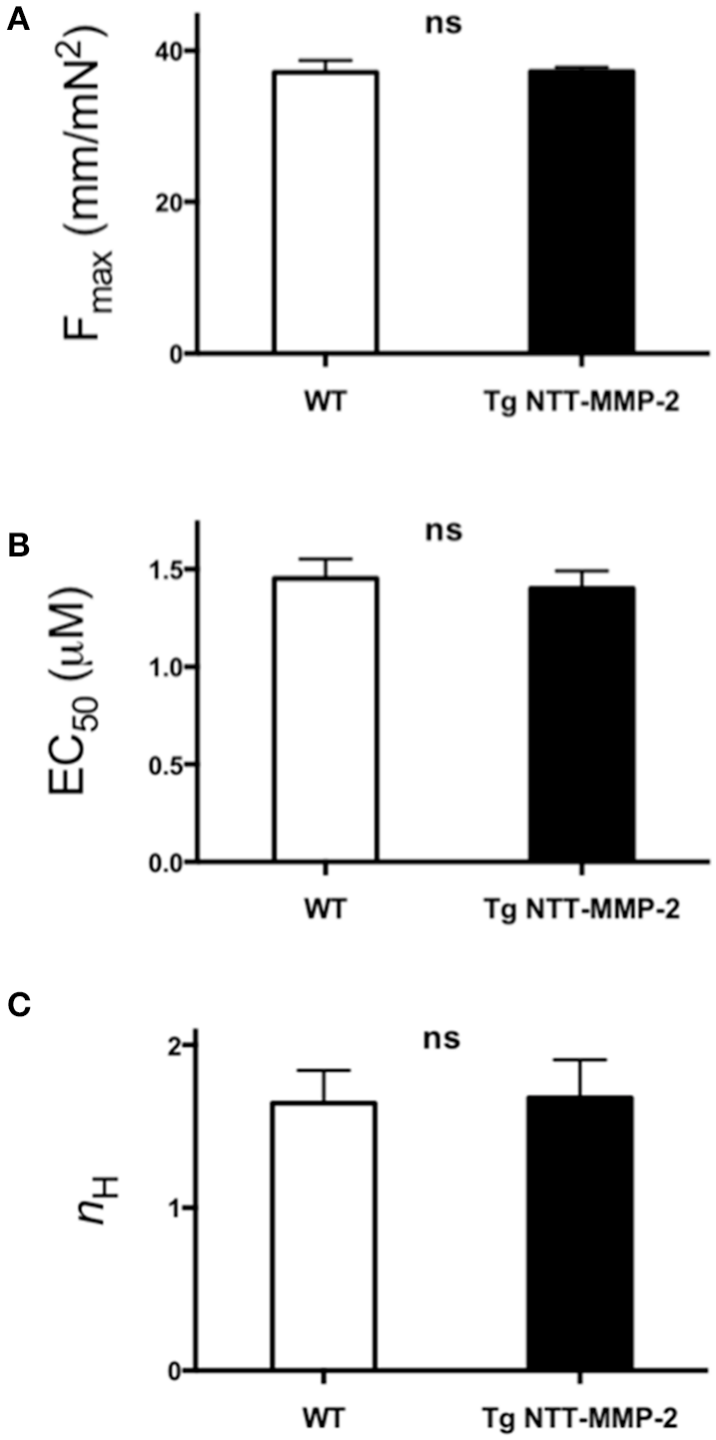
**Pooled data of Hill parameters derived from fitting the force-Ca^2+^ relationship for individual experiments to the Hill equation. (A)** F_max_ (maximum Ca^2+^-activated force). **(B)** Ca^2+^ sensitivity EC_50_ ([Ca^2+^] at 50% Fmax). **(C)** Hill coefficient *n*_H_ (slope of Ca^2+^-force relationship at EC_50_). For all Hill parameters, no statistical differences were found between NTT-MMP-2 vs. wild type (*P* ns, Two-Way ANOVA; mean ± SE, *n* = 4/group).

These same trabeculae were chemically skinned and exposed to activating solutions with various levels of free [Ca^2+^] to measure myofilament force, separate from influences from the muscle activation system or muscle energy stores. Figure 3B shows that myofilament force development by trabeculae from NTT-MMP-2 hearts was indistinguishable from that of trabeculae from wild type hearts. Furthermore, Hill parameters calculated by fitting myofilament force-Ca^2+^ relationships to the Hill equation were not different between NTT-MMP-2 and wild type (Figure 4).

The data from skinned myocardium suggests that the decreased force development of intact NTT-MMP-2 trabeculae (Figure 3A) did not involve intrinsically impaired myofilament function. This conclusion is consistent with the histology data showing that NTT-MMP-2 did not localize to the myofilaments (Figure 2). The absence of an effect of NTT-MMP-2 on myofilament force suggests that NTT-MMP-2 reduced the active force development of intact muscle by impairing other processes required for force generation such as impaired muscle activation.

These findings with NTT-MMP-2 expression differ considerably from those obtained with FL-MMP-2 expression, which was associated with markedly reduced myofilament force development (Wang et al., 2006). This suggests that there are distinctly different mechanisms involved in the reductions of myocardial force mediated by NTT-MMP expression vs. FL-MMP-2 expression. With FL-MMP-2 reducing myofilament contraction, but NTT-MMP-2 not affecting myofilament contraction.

### Transgenic NTT-MMP-2 expression reduced myocyte Ca^2+^ transients

**Figure 5:**
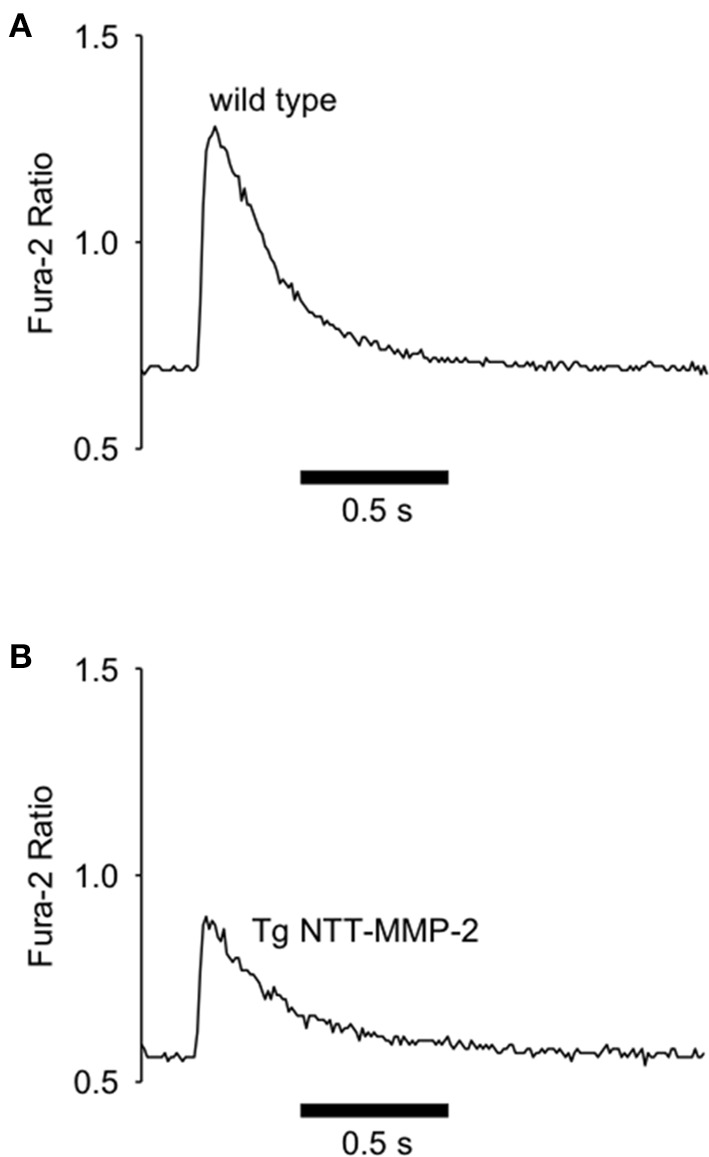
**Reduced Ca^2+^ transients with NTT-MMP-2 expression**. Original records of Fura-2 Ca^2+^ transients recorded from electrically stimulated single cardiac myocytes. Compared to wild type controls **(A)**, NTT-MMP-2 transgenic cells had reduced Ca^2+^ transient amplitude **(B)**.

**Figure 6:**
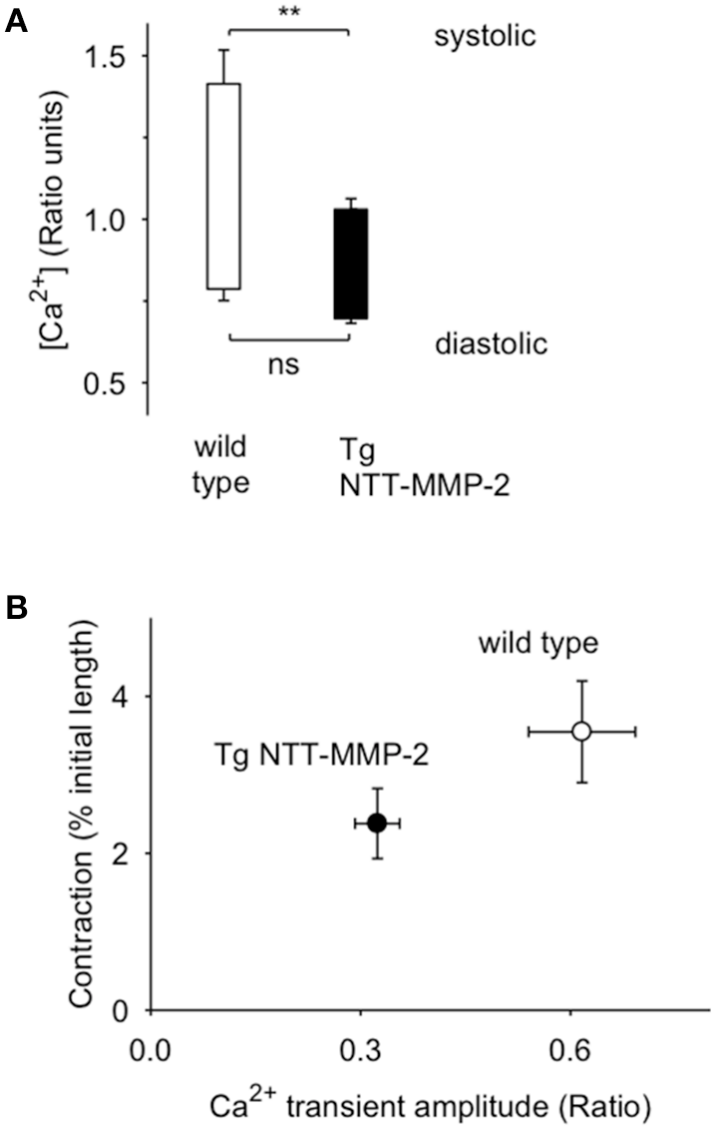
**Pooled data from cells from Tg NTT-MMP-2 mice and wild type. (A)** Cells from Tg NTT-MMP-2 hearts had lower systolic Ca^2+^ levels than cells from wild type (^**^P < 0.01). **(B)** Pooled data (mean ± SE) for contraction amplitude vs. Ca^2+^ transient amplitude for cells from Tg NTT-MMP-2 hearts and wild type [*n* = 16.22 cells/group from wild type mice (*n* = 5) and NTT-MMP-2 expressing mice (*n* = 4)]. Cells from Tg NTT-MMP-2 hearts had smaller Ca^2+^ transients and contractions compared to wild type. There was a significant linear relation between contraction vs. Ca^2+^ transient amplitude (*P* < 0.001).

We recorded Ca^2+^ transients in electrically stimulated single cardiac myocytes using the fluorescent Ca^2+^ indicator Fura-2. Figure 5 shows Ca^2+^ transients recorded from single myocytes from wild type or NTT-MMP-2 transgenic hearts. The myocyte Ca^2+^ transient amplitude was appreciably reduced by transgenic NTT-MMP-2 expression compared to wild type. The pooled data show that the amplitude of the Ca^2+^ transient was reduced approximately 50% in NTT-MMP-2 myocytes vs. wild type myocytes (Figure 6A). The reduced Ca^2+^ transient caused by NTT-MMP-2 expression was due to a reduced systolic Ca^2+^ level without appreciable effect on diastolic Ca^2+^ level. Consistent with a similar diastolic Ca^2+^ level, the diastolic sarcomere length was identical in NTT-MMP-2 expressing myocytes (1.80 ± 0.01 μm, *n* = 16) vs. wild type myocytes (1.80 ± 0.01 μm, *n* = 22). The pooled data show that for myocytes expressing NTT-MMP-2, the amplitude of the Ca^2+^ transient and contractions were reduced compared to myocytes from wild type hearts (Figure 6B).

NTT-MMP-2 expression did not have an appreciable effect on the dynamics of contraction and relaxation of cardiac myocytes. For NTT-MMP-2 mice vs. wild type controls, the time to peak contraction and time to 50% relaxation were: 92 ± 8 ms, and 90 ± 7 ms, *n* = 19; vs. 95 ± 5 ms and 100 ± 10 ms, *n* = 22; there was not a significant difference in these values between NTT-MMP-2 and wild type (*P* > 0.05, ANOVA).

In summary, the data show that unlike FL-MMP-2, NTT-MMP-2 was not localized with the myofilaments, and did not cause impaired myofilament force development. NTT-MMP-2 caused impaired muscle activation, evidenced by decreased Ca^2+^ transients.

## Discussion

The major findings of this study were that transgenic expression of NTT-MMP-2 impaired myocardial contraction, and that impaired contraction did not involve decreased myofilament force. Instead, NTT-MMP-2 expression was associated with decreased calcium transients. These findings are in contrast to our previous findings that FL-MMP-2 expression resulted in decreased myocardial contraction that did involve decreased myofilament force. Thus, the present study expands our understanding of the pathobiology of MMP-2 by demonstrating that two distinct isoforms of MMP-2 impair different intracellular processes.

Underscoring the disease relevance of MMP-2-mediated cardiac injury, studies in humans have implicated MMP-2 in LV dysfunction and dysfunctional remodeling after myocardial infarction (Nilsson et al., 2011; Cogni et al., 2013). Previous studies report decreased *in-vivo* function due to transgenic expression of FL-MMP-2 (Bergman et al., 2007) or NTT-MMP-2 (Lovett et al., 2013). Moreover, hemodynamic function measured *in-vitro* after ischemic injury showed reduced left ventricular developed pressure with transgenic expression of FL-MMP-2 (Zhou et al., 2007) or NTT-MMP-2 (Lovett et al., 2013).

FL-MMP-2 was confirmed to localize to the myofilaments (Bergman et al., 2007), consistent with a known effect of FL-MMP-2 causing myofilament injury (Wang et al., 2006). In contrast, NTT-MMP-2 did not localize to myofilaments, and did not cause myofilament injury. Previously, NTT-MMP-2 was found to localize to mitochondria primarily within the subsarcolemmal space, and to perpendicular arrays across the long axis of individual cardiomyocytes (Lovett et al., 2013). The present study confirmed this and found that NTT-MMP-2 appeared within clusters along the Z-line.

These studies used transgenic NTT-MMP-2 mice at 4–5 months of age. At this age these mice have normal cardiac histology. Physiologic characterization of these mice with serial echocardiography revealed normal functional parameters other than a small decrease in left ventricular ejection fraction (Lovett et al., 2013). Thus, the impaired contractile function defined in the current study precedes the development of severe systolic dysfunction and is not a consequence thereof.

FL-MMP-2 is known to be involved in regulation of the cardiac extracellular matrix (Spinale, 2007), and to contribute to dysfunctional extracellular remodeling in the setting of myocardial infarction (Hayashidani et al., 2003). However, recent studies have recognized that FL-MMP-2 is also an intracellular protease (Schulz, 2007), with almost 40% of newly synthesized FL-MMP-2 retained in the cytosol due to an inefficient secretory signal (Ali et al., 2012). In the setting of oxidative stress, intracellular FL-MMP-2 is activated by release of the inhibitory prodomain, leading to FL-MMP-2-mediated cleavage of sarcomeric proteins with resultant contractile dysfunction (Schulz, 2007).

NTT-MMP-2 is a novel intracellular MMP-2 isoform generated by oxidative stress-mediated activation of an alternate promoter in the first intron of the MMP-2 gene (Lovett et al., 2012, 2013). NTT-MMP-2 lacks the secretory peptide and the inhibitory prodomain and thus is enzymatically active and acts exclusively on intracellular targets. Like FL-MMP-2, cardiac-specific NTT-MMP-2 transgene expression also leads to systolic failure (Lovett et al., 2013). But the mechanisms involved have been unclear.

In contrast to FL-MMP-2, which localizes to the myofilaments, NTT-MMP-2 is not localized to the myofilaments. The absence of NTT-MMP-2 on the myofilaments is consistent with our finding that myofilament force was not impaired in NTT-MMP-2 transgenic hearts. Intact myocardium from hearts with transgenic expression of NTT-MMP-2 had a 50% reduction in electrically stimulated myocardial force. Therefore, the reduced force of intact myocardium was not due to a reduction of intrinsic myofilament force. Instead, we found that NTT-MMP-2 was localized to subsarcolemmal mitochondria and to clusters that were in register with the Z-line of the sarcomere. NTT-MMP-2 localized to the Z-line could reflect association with the transverse tubule (t-tubule), which is critically involved in excitation-contraction coupling. Disruptions of t-tubular structure and function have been implicated with impaired myocyte activation in heart failure (Cannell et al., 2006; Orchard and Brette, 2008). NTT-MMP-2 effects on the t-tubule might be consistent with our finding that NTT-MMP-2 expression substantially impaired the amplitude of the Ca^2+^ transient in isolated myocytes. Impaired muscle activation with decreased Ca^2+^ transients may play a role in the reduced myocardial contraction caused by NTT-MMP-2 expression.

A prominent ultrastructural feature of the NTT-MMP-2 transgenic mice was the presence of swollen mitochondria with loss of organized cristae (Lovett et al., 2013). These morphologic features are characteristic of the opening of the mitochondrial permeability transition pore (MTP) (Buki et al., 2000). MTP opening is associated with enhanced oxidative stress due to uncoupling of the mitochondrial electron transport chain (Halestrap and Pasdois, 2009). Cardiac oxidant stress has multiple deleterious effects on calcium and sodium handling, which may also contribute to impaired contraction and Ca^2+^ transients associated with NTT-MMP-2 expression (Sag et al., 2013).

### Limitations

We studied contracting cells and trabeculae, and demembranated trabeculae at 22°C. While demembranated trabeculae are more stable for study at low temperature, nevertheless, temperature is known to affect contraction and relaxation properties (Janssen et al., 2002). Although wild type and transgenic groups were studied under the same conditions, caution is needed for extrapolating from the results of this study to the intact heart. Furthermore, to explore the full range of muscle activation, we used high bath Ca^2+^ levels in both intact and skinned myocardium. High Ca^2+^ levels and maximal levels of activation are not achieved *in-vivo* and therefore, caution is needed for extrapolating from these experimental conditions. To understand the basis of decreased Ca^2+^ transients with transgenic NTT-MMP-2 expression, mechanistic studies are needed to investigate the roles of mitochondria and Ca^2+^-handling systems that might be affected by NTT-MMP-2. Finally, as NTT-MMP-2 is activated by redox stress, greater understanding of the effect of NTT-MMP-2 expression in the context of redox stress is needed.

## Conclusion

Two discrete MMP-2 isoforms have distinctive pathophysiological mechanisms that impair cardiomyocyte contractility. FL-MMP-2 was previously reported to injure the myofilaments, in contrast, the current study found NTT-MMP-2 does not injure the myofilaments but impairs muscle activation involving reduced Ca^2+^ transients.

### Conflict of interest statement

The authors declare that the research was conducted in the absence of any commercial or financial relationships that could be construed as a potential conflict of interest.

## References

[B1] Alfonso-Jaume M. A. Bergman M. R. Mahimkar R. Cheng S. Jin Z. Q. Karliner J. S. (2006). Cardiac ischemia-reperfusion injury induces matrix metalloproteinase-2 expression through the AP-1 components FosB and JunB. Am. J. Physiol. Heart Circ. Physiol. 291, H1838–H1846 10.1152/ajpheart.00026.2006 16699069

[B2] Ali M. A. Chow A. K. Kandasamy A. D. Fan X. West L. J. Crawford B. D. (2012). Mechanisms of cytosolic targeting of matrix metalloproteinase-2. J. Cell. Physiol. 227, 3397–3404 10.1002/jcp.24040 22212960

[B3] Ali M. A. Fan X. Schulz R. (2011). Cardiac sarcomeric proteins: novel intracellular targets of matrix metalloproteinase-2 in heart disease. Trends Cardiovasc. Med. 21, 112–118 10.1016/j.tcm.2012.03.008 22681966

[B4] Bergman M. R. Teerlink J. R. Mahimkar R. Li L. Zhu B. Q. Nguyen A. (2007). Cardiac matrix metalloproteinase-2 expression independently induces marked ventricular remodeling and systolic dysfunction. Am. J. Physiol. Heart Circ. Physiol. 292, H1847–H1860 10.1152/ajpheart.00434.2006 17158653

[B5] Buki A. Okonkwo D. O. Wang K. K. Povlishock J. T. (2000). Cytochrome c release and caspase activation in traumatic axonal injury. J. Neurosci. 20, 2825–2834 10751434 10.1523/JNEUROSCI.20-08-02825.2000 PMC6772193

[B6] Cannell M. B. Crossman D. J. Soeller C. (2006). Effect of changes in action potential spike configuration, junctional sarcoplasmic reticulum micro-architecture and altered t-tubule structure in human heart failure. J. Muscle Res. Cell Motil. 27, 297–306 10.1007/s10974-006-9089-y 16897575

[B7] Chu C. Thai K. Park K. W. Wang P. Makwana O. Lovett D. H. (2013). Intraventricular and interventricular cellular heterogeneity of inotropic responses to alpha(1)-adrenergic stimulation. Am. J. Physiol. Heart Circ. Physiol. 304, H946–H953 10.1152/ajpheart.00822.2012 23355341 PMC3625891

[B8] Cogni A. L. Farah E. Minicucci M. F. Azevedo P. S. Okoshi K. Matsubara B. B. (2013). Metalloproteinases-2 and -9 predict left ventricular remodeling after myocardial infarction. Arq. Bras. Cardiol. 100, 315–321 10.5935/abc.20130049 23525272

[B9] Halestrap A. P. Pasdois P. (2009). The role of the mitochondrial permeability transition pore in heart disease. Biochim. Biophys. Acta. 1787, 1402–1415 10.1016/j.bbabio.2008.12.017 19168026

[B10] Hayashidani S. Tsutsui H. Ikeuchi M. Shiomi T. Matsusaka H. Kubota T. (2003). Targeted deletion of MMP-2 attenuates early LV rupture and late remodeling after experimental myocardial infarction. Am. J. Physiol. Heart Circ. Physiol. 285, H1229–H1235 10.1152/ajpheart.00207.2003 12775562

[B11] Janssen P. M. Stull L. B. Marban E. (2002). Myofilament properties comprise the rate-limiting step for cardiac relaxation at body temperature in the rat. Am. J. Physiol. Heart Circ. Physiol. 282, H499–H507 10.1152/ajpheart.00595.2001 11788397

[B12] Lovett D. H. Mahimkar R. Raffai R. L. Cape L. Maklashina E. Cecchini G. (2012). A novel intracellular isoform of matrix metalloproteinase-2 induced by oxidative stress activates innate immunity. PLoS ONE 7:e34177 10.1371/journal.pone.0034177 22509276 PMC3317925

[B13] Lovett D. H. Mahimkar R. Raffai R. L. Cape L. Zhu B. Q. Jin Z. Q. (2013). N-terminal truncated intracellular matrix metalloproteinase-2 induces cardiomyocyte hypertrophy, inflammation and systolic heart failure. PLoS ONE 8:e68154 10.1371/journal.pone.0068154 23874529 PMC3712965

[B14] Mukherjee R. Mingoia J. T. Bruce J. A. Austin J. S. Stroud R. E. Escobar G. P. (2006). Selective spatiotemporal induction of matrix metalloproteinase-2 and matrix metalloproteinase-9 transcription after myocardial infarction. Am. J. Physiol. Heart Circ. Physiol. 291, H2216–H2228 10.1152/ajpheart.01343.2005 16766634

[B15] Nilsson L. Hallen J. Atar D. Jonasson L. Swahn E. (2011). Early measurements of plasma matrix metalloproteinase-2 predict infarct size and ventricular dysfunction in ST-elevation myocardial infarction. Heart 98, 31–36 10.1136/heartjnl-2011-300079 21727201

[B16] O'Connell T. D. Rodrigo M. C. Simpson P. C. (2007). Isolation and culture of adult mouse cardiac myocytes. Methods Mol. Biol. 357, 271–296 10.1385/1-59745-214-9:271 17172694

[B17] Orchard C. Brette F. (2008). t-Tubules and sarcoplasmic reticulum function in cardiac ventricular myocytes. Cardiovasc. Res. 77, 237–244 10.1093/cvr/cvm002 18006490

[B18] Sag C. M. Wagner S. Maier L. S. (2013). Role of oxidants on calcium and sodium movement in healthy and diseased cardiac myocytes. Free Radic. Biol. Med. 63, 338–349 10.1016/j.freeradbiomed.2013.05.035 23732518

[B19] Sawicki G. Leon H. Sawicka J. Sariahmetoglu M. Schulze C. J. Scott P. G. (2005). Degradation of myosin light chain in isolated rat hearts subjected to ischemia-reperfusion injury: a new intracellular target for matrix metalloproteinase-2. Circulation 112, 544–552 10.1161/CIRCULATIONAHA.104.531616 16027249

[B20] Schulz R. (2007). Intracellular targets of matrix metalloproteinase-2 in cardiac disease: rationale and therapeutic approaches. Annu. Rev. Pharmacol. Toxicol. 47, 211–242 10.1146/annurev.pharmtox.47.120505.105230 17129183

[B21] Spinale F. G. (2007). Myocardial matrix remodeling and the matrix metalloproteinases: influence on cardiac form and function. Physiol. Rev. 87, 1285–1342 10.1152/physrev.00012.2007 17928585

[B22] Wang G. Y. Bergman M. R. Nguyen A. P. Turcato S. Swigart P. M. Rodrigo M. C. (2006). Cardiac transgenic matrix metalloproteinase-2 expression directly induces impaired contractility. Cardiovasc. Res. 69, 688–696 10.1016/j.cardiores.2005.08.023 16183043

[B23] Wang W. Schulze C. J. Suarez-Pinzon W. L. Dyck J. R. Sawicki G. Schulz R. (2002). Intracellular action of matrix metalloproteinase-2 accounts for acute myocardial ischemia and reperfusion injury. Circulation 106, 1543–1549 10.1161/01.CIR.0000028818.33488.7B 12234962

[B24] Zhou H. Z. Ma X. Gray M. O. Zhu B. Q. Nguyen A. P. Baker A. J. (2007). Transgenic MMP-2 expression induces latent cardiac mitochondrial dysfunction. Biochem. Biophys. Res. Commun. 358, 189–195 10.1016/j.bbrc.2007.04.094 17475219 PMC3423089

